# Case report: olaparib use in metastatic lung adenocarcinoma with *BRCA2* pathogenic variant

**DOI:** 10.1101/mcs.a006223

**Published:** 2022-12

**Authors:** Jonathan Soon Jian Hao, Chan Sock Hoai, Daniel Tan Shao Weng, Joanne Ngeow, Jianbang Chiang

**Affiliations:** 1 Cancer Genetics Service, National Cancer Centre Singapore, Singapore 169610, Singapore;; 2 Division of Medical Oncology, National Cancer Centre Singapore, Singapore 169610, Singapore;; 3 Duke-NUS Medical School, Singapore 169857, Singapore;; 4 Lee Kong Chian School of Medicine, Nanyang Technological University, Singapore 308232, Singapore;; 5 Institute of Molecular and Cellular Biology, Agency for Science, Technology and Research, Singapore 138673, Singapore

**Keywords:** chronic lung disease

## Abstract

Poly (ADP-ribose) polymerase (PARP) inhibitors have been approved in malignancies associated with germline *BRCA1* or *BRCA2* pathogenic variants, such as breast, ovarian, prostate, and pancreatic cancer. In malignancies not associated with germline *BRCA1* or *BRCA2* pathogenic variants, the therapeutic relevance of PARP inhibitors is less clear. Non-small-cell lung cancer (NSCLC) is known to demonstrate somatic alterations in *BRCA1* or *BRCA2* gene. The current report is on a gentleman with metastatic lung adenocarcinoma with a somatic *BRCA2* pathogenic variant, who was effectively treated with olaparib. Furthermore, we discuss the existing data for use of PARP inhibitors in NSCLC. This study highlights the utility of next-generation sequencing in identifying gene mutations and demonstrates how such information can be used to select targeted therapies in patients with actionable molecular alterations.

## INTRODUCTION

Since the discovery of oncogenic driver mutations, the therapeutic landscape of non-small-cell lung cancer (NSCLC) has evolved exponentially. Genomic profiling has set the stage for personalized cancer therapy. NSCLC is known to demonstrate somatic alterations in the *BRCA1/2* gene and homologous recombination repair (HRR) genes such as *ATM*, *FANCA*, and *PALB2* (Lord and Ashworth 2016). Based on The Cancer Genome Atlas data set, the rates of *BRCA1* or *BRCA2* variants in lung adenocarcinoma were 3.0% and 4.8%, respectively. (Cancer Genome Atlas Research Network 2014).

Poly (ADP-ribose) polymerase (PARP) inhibitors trap PARP on DNA at sites of single strand breaks, preventing cellular DNA repair machinery from correcting the initial insult. HRR is necessary to resolve these PARP–DNA interactions via double-stranded break repair. Double-stranded break repair are defective in homologous recombination–deficient (HRD) cancer cells with *BRCA1 or BRCA2* pathogenic variants (PVs). PARP inhibitors confer synthetic lethality to these cells with accumulation of DNA damage causing cell death. PARP inhibitors are approved in malignancies associated with germline *BRCA1/2* PV such as breast, ovarian, prostate, and pancreatic cancer. The therapeutic relevance of olaparib remains less defined in NSCLC and other nongermline *BRCA1*/*2* related malignancies.

A preclinical study (Ji et al. 2020) depleted *BRCA1*/*2* in a NSCLC cell line and found that cells lacking homologous recombination (HR) proteins were hypersensitive to olaparib. The authors propose that olaparib induces apoptosis in HR-deficient NSCLC cells. Two phase II trials (Fennell et al. 2020; Postel-Vinay et al. 2021) compared olaparib versus placebo monotherapy in patients with metastatic NSCLC. Both trials showed no progression-free survival (PFS) benefit or overall survival (OS) benefit with olaparib.

With this report, we discuss the case of a 64-yr-old male patient with metastatic lung adenocarcinoma with a somatic *BRCA2* PV, whose treatment with olaparib led to a clinical and radiologic response of his disease.

## CLINICAL PRESENTATION

The patient was a 64-yr-old Chinese gentleman with a smoking history of forty pack-years and hypertension who presented with a skin lump over the epigastrium in March 2019. Significant family history included his sister who was diagnosed with breast cancer in her 40s and had undergone surgery; further details were unavailable. Excision of the lump showed an adenocarcinoma strongly positive for CK7, TTF-1, and Napsin A. Computed tomography (CT) of the chest, abdomen, and pelvis showed a spiculated left upper lobe nodule with mediastinal lymphadenopathy and left adrenal metastases. Molecular testing on the lump revealed epidermal growth factor (*EGFR*) mutation c.2126A > C(p.Glu709Ala) and c.2156_2157delins CA(p.Gly719Ala). No disruptions were seen in the *ALK* and *ROS1* genes, and the ratio of MET to CEP7 signals was 1.1. Programmed death ligand-1 (PD-L1) tumor proportion score (TPS) by immunohistochemistry was 40%.

First-line treatment using afatinib 40 mg once daily commenced in April 2019 with partial response. A restaging CT scan 2 months later showed new lytic lesions in the left femur and left ulna. In view of pain at the left thigh and forearm and risk of fracture, the patient underwent a prophylactic left femur intramedullary nail insertion and left ulna plating in August 2019. Postoperatively, five fractions of radiotherapy were administered to both sites at 20 Gy each. Afatinib was restarted after radiotherapy and continued until progression in March 2020, with a disease control of 11 mo.

**Figure 1.**
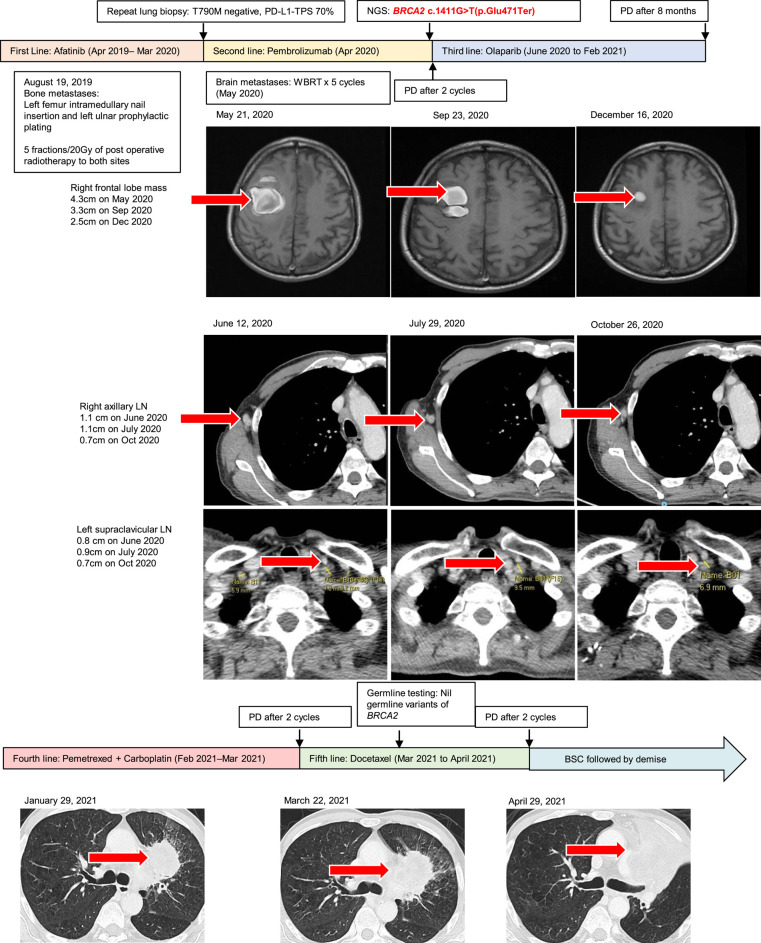
Metastatic non-small-cell lung cancer (NSCLC) treatment using different regimens and results of next-generation sequencing (NGS). (BSC) Best supportive care, (PD) progression of disease, (PDL-1 TPS) programmed death-ligand 1 tumor proportion score, (WBRT) whole brain radiotherapy.

In December 2019, a liquid biopsy was performed to look for T790M mutation, which was negative. In March 2020, a lung biopsy was performed on the lung primarily to look for T790M mutation, which was also negative. The lung biopsy showed a PD-L1 TPS score of 70%. Second-line pembrolizumab was started in April 2020. In May 2020, after two cycles of pembrolizumab, the patient had complained of left upper and lower limb weakness. A magnetic resonance imaging (MRI) brain scan showed multiple brain metastases, with the largest in the right frontal lobe (Fig. 1). The patient underwent five fractions of whole brain radiotherapy (WBRT) at 20 Gy.

In May 2020, next-generation sequencing (NGS) (Oncomine Comprehensive Assay v3) was conducted on the lung biopsy, which revealed a somatic *BRCA2* c.1411G > T (p.Glu471Ter) pathogenic variant. The patient was discussed at a molecular multidisciplinary tumor board in a tertiary cancer center and deemed to be suitable for PARP inhibitors (Seet et al. 2021). A baseline CT of chest, abdomen, and pelvis in June 2020 showed mixed response, with increase in axillary lymphadenopathy, stable disease in the mediastinal and supraclavicular lymphadenopathy, and decrease in size of lung primary and skin metastases. Olaparib was then given from June 2020 at a dose of 300 mg twice a day. A CT scan in July 2020 showed stable disease in lymph nodes, bone, and skin. A partial response was then achieved, with an MRI of the brain in both September 2020 and December 2020 showing reduction in the size of the brain metastases (Fig. 1). A CT scan of the chest, abdomen, and pelvis in October 2020 showed reduction in the size of the supraclavicular and axillary lymphadenopathy (Fig. 1), and stable disease in the lung primary and hilar lymphadenopathy.

A CT scan in January 2021 showed progression of the primary lung tumor and increasing lymphadenopathy, with a PFS of 8 mo while on olaparib. He was switched to pemetrexed-carboplatin. Two cycles later, his disease in the lung, lymph nodes, adrenals, and bone progressed. He was then switched to docetaxel. The patient was on best supportive care since May 2021 and died in June 2021.

## GENOMIC ANALYSIS

The specimen obtained from the epigastric lump was sent for *EGFR* mutation analysis via direct Sanger sequencing and *ALK, ROS1*, *RET*, and *MET/CEP7* analyses by interphase fluorescence in situ hybridization (FISH); details are described in the Methods section. After progression on afatinib, peripheral blood and the biopsy of the lung primary was sent for *EGFR* mutation analysis to look for a *EGFR* T790M mutation, which would affect the choice of *EGFR* tyrosine kinase inhibitor.

The specimen from the lung biopsy was sent for NGS panel to guide further treatment. The tumor percentage of the sequenced biopsy was 70%. Details of the sequencing are presented in Table 1. Analysis of the tumor showed a somatic *BRCA2* c.1411G > T(p.Glu471Ter) variant with variant frequency of 28.4%, an *RB1* c.1807G > A(p.Ala603Thr) variant with variant frequency of 35.6%, and mean estimated tumor mutation burden of 14 Muts/Mb. The two *EFGR* mutations found on initial molecular testing were similarly demonstrated in the NGS panel. Three variants of unknown significance in the *ATR*, *CREBBP*, and *SLX4* genes were found; variant allele frequencies were not provided. No fusions, amplifications, nor copy number variations were detected.

**Table 1.** Genomic findings and their variant interpretation identified on somatic sequencing of lung primary

Gene	Chromosome	HGVS DNA reference	HGVS protein reference	Variant type	Predicted effect	dbSNP/dbVar ID	Variant allele frequency (%)
*BRCA2*	Chr 13	c.1411G > T (NM_000059.4)	p.Glu471Ter	Substitution	Nonsense	Rs80358428	28.8
*RB1*	Chr 13	c.1807G > A (NM_000321.3)	p.Ala603Thr	Substitution	Missense	Rs777791058	35.6
*EGFR*	Chr 7	c.2126A > C (NM_005228)	p.Glu709Ala	Substitution	Missense	Rs397517085	28.4
*EGFR*	Chr 7	c. 2156G_2157delinsCA (NM_005228)	p.Gly719Ala	Deletion-insertion	Missense	N/A	27.8
*ATR*	Chr 3	c.5386A > C (NM_001184)	p.Lys1796Gln	Substitution	Missense	N/A	Not provided by lab
*CREBBP*	Chr 16	c.2239A > G (NM_004380)	p.Met747Val	Substitution	Missense	N/A	Not provided by lab
*SLX4*	Chr 16	c.4765C > T (NM_032444)	p.Arg1589Cys	Substitution	Missense	Rs181782315	Not provided by lab

(HGVS) Human Genome Variation Society.

**Figure 2.**
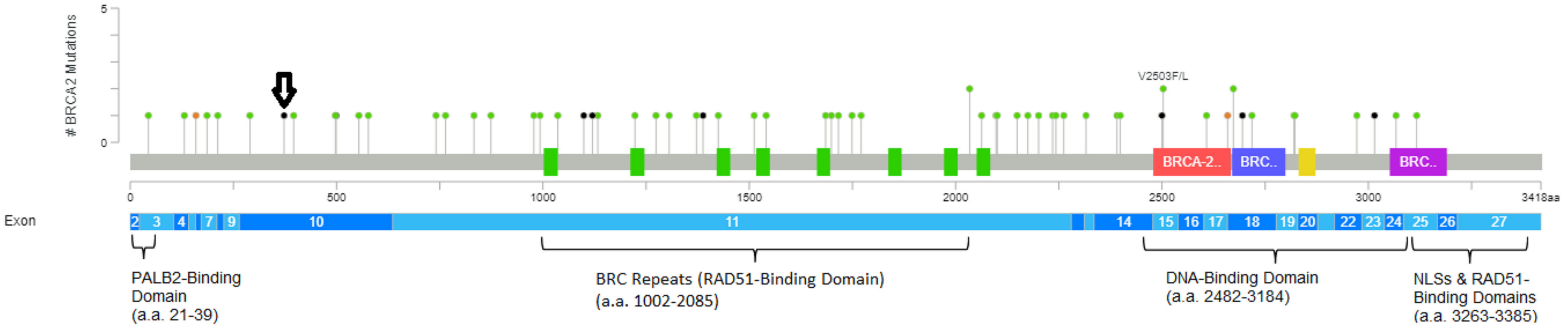
Lollipop plot of all reported mutations in *BRCA2* among a total of 1144 sequenced non-small-cell lung cancers from The Cancer Genome Atlas Pan-Lung Cancer data set. (Green) missense mutation, (black) truncating mutation, (orange) splice mutation. These include the present case (lollipop with the black arrow) that contains a likely pathogenic *BRCA2* variant.

In the present case, the G > T variant within *BRCA2* at genome coordinate [GRCh37] Chr 13:32907026 is predicted to result in an amino acid substitution of glutamic acid with a stop codon, causing a nonsense variant that leads to loss of normal protein function. Loss-of-function variants in *BRCA2* are known to be pathogenic (Mesman et al. 2020). The absence of *BRCA2* leads to defective DNA double-strand break repair by homologous recombination, increasing the risk of tumorigenesis and susceptibility to PARP inhibitors. Figure 2 illustrates the lollipop plot of *BRCA2* mutations in an NSCLC cohort of 1144 patients from The Cancer Genome Atlas Pan-Lung Cancer data set. This has been edited to include our patient's variant, which was not observed in prior NSCLC cohorts (Campbell et al. 2016).

Germline testing performed on the patient's blood in March 2021 showed *EGFR* c.3353C > T(p.Ala1118Val) variant of uncertain significance, but no germline *BRCA2* PV was found. Details of the germline testing are found in Table 2. It did not find other genes associated with hereditary breast and ovarian cancer such as *ATM, CHEK2, PALB2, RAD51C*, and *RAD51D*. Based on the laboratory interpretation, algorithms developed to predict the effect of this missense change on protein structure and function (SIFT, PolyPhen-2, Align-GVGD) all suggest that the *EGFR* variant is likely to be tolerated, but these predictions had not been confirmed by functional studies and their clinical significance is uncertain.

**Table 2.** Genomic findings and their variant interpretation identified on germline testing of peripheral blood

Gene	Chromosome	HGVS DNA reference	HGVS protein reference	Variant type	Predicted effect	dbSNP/dbVar ID	Variant allele frequency (%)
*EGFR*	Chr 7	c.3353C > T (NM_005228)	p.Ala1118Val	Substitution	Missense	Rs773996588	Not provided by lab

(HGVS) Human Genome Variation Society.

## DISCUSSION

In this case report, we showed the clinical efficacy of olaparib in a patient with metastatic NSCLC harboring a somatic *BRCA2* PV and germline *BRCA2* wild type. The patient's PFS of 8 mo with olaparib was superior to pembrolizumab and pemetrexed-carboplatin. Although the partial response in the brain may be attributed to the effects of the WBRT, the disease control in the lymph nodes is likely due to the PARP inhibitor. These observations may support the role of extended genomic testing in this patient population. Multiple DNA repair genes in the HRR pathway may be associated with PARP inhibitor sensitivity. Not every patient with DNA repair defects will have clinical benefit with PARP inhibition, and there are ongoing efforts to identify biomarkers that will predict response to PARP inhibitors (Lord and Ashworth 2016). To date, there are only case reports that describe the benefit of PARP inhibitors in metastatic NSCLC, but these reports were in patients harboring a germline *BRCA1/2* PV (Talreja et al. 2020; Zhang et al. 2021; Wu et al. 2022).

Given the tumor percentage of 70% and a variant allele frequency of 28.4%, it is presumed that the patient's *BRCA2* PV is a monoallelic *BRCA2* mutation. The NGS panel used did not provide information about the allelic status or the HRD status of the tumor. Biallelic alterations of HRR genes are significantly associated with genomic features of homologous recombination deficiency, whereas mono-allelic alterations are not (Riaz et al. 2017). Apart from looking at mutations in genes involved in the HRR pathway, the HRD status may also be determined by evaluating the effect of genomic scarring. Genomic scars are aberrations that result in structural changes in the chromosomes. The most relevant genomic scars include the loss of heterozygosity (LOH), telomeric imbalance (TAI), and large-scale transitions (LSTs) (Lord and Ashworth 2016). When measured together, this produces a genomic instability score that may be used as an indicator of HRD status and maximize the identification of samples with HRD (Marquad et al. 2015). Germline testing of our patient did not identify any pathogenic variants of HRR genes that may have resulted in biallelic alterations. In our patient who had a somatic *BRCA2* pathogenic variant, LOH of the wild-type allele would have resulted in a biallelic alteration and explain the response to olaparib. Therefore, it would have been interesting to run a larger sequencing panel such as the Trusight Oncology 500 HRD panel, which could provide more information on the presence of genomic scars such as LOH.

The Oncomine Comprehensive Assay v3 NGS panel, an amplicon-based assay, has its own limitations. The amplicon-based approach relies on primers that flank the interest of sequencing. This can lead to false negatives because of allele dropout or genomic deletions (Ionescu et al. 2022). A study showed that amplicon-based assays had a lower rate of detecting gene fusions in NSCLC patients when compared to hybrid capture–based assays (Heydt et al. 2021). Another study showed that commercially developed amplicon assays were limited in the detection of *MET* exon 14 skipping events in NSCLC patients (Poirot et al. 2017).

The response to olaparib runs contrary to the results from the two prior phase II studies that have looked at PARP inhibitors in platinum sensitive advanced NSCLC (Fennell et al. 2020; Postel-Vinay et al. 2021). In both trials no genomic sequencing was performed to identify patients with *BRCA1/2* PVs. Only platinum sensitivity was hypothesized to enrich for patients who may have underlying HRR deficiency such as *BRCA1/2* PV and therefore be sensitive to olaparib. Yet, platinum sensitivity may not be sufficient to rule out clinical benefit from PARP inhibitors. Unlike PARP inhibitor sensitivity, which results from defective DNA single-stranded break repair, platinum sensitivity may result from defective nucleotide excision repair (Ceccaldi et al. 2016). In addition, first-line chemotherapy for NSCLC is platinum-based. In the trial by Fennell et al. they concluded that design parameters justifying a phase III trial were met in the unadjusted PFS analysis, with a trend toward a longer PFS and OS in the olaparib arm of the study. This highlights the need for further translational studies to investigate the possibilities of olaparib use in patients with platinum sensitive NSCLC.

In our case, we observed an initial response to PARP inhibition followed by progression of an aggressive tumor resistant to platinum-based chemotherapy. Overlapping mechanisms of resistance to platinum and PARP inhibitor treatment may be the reason for the patient's poorer response to pemetrexed-carboplatin. Restoration of the functional HRR pathway in a HRD tumor may occur via *BRCA1/2* or HR gene reversion mutation which functionally restores protein activity (Lord and Ashworth 2016). These mechanisms of resistance are alluded to in the post-hoc analysis of the SOLO2 trial, in which patients with epithelial ovarian cancer who received maintenance olaparib had marked reduction in the efficacy of platinum-based chemotherapy as seen in the time to second progression (Frenel et al. 2020). A limitation of our research includes not performing a biopsy on the relapsed, progressive tumor to investigate the reasons for developing resistance to PARP inhibitors and platinum chemotherapy.

According to the American College of Medical Genetics and Genomics (ACMG) guidelines, any patient with a known pathogenic or likely pathogenic variant in *BRCA1/2* in any tumor type should be further investigated with germline testing to look for germline mutations (Kalia et al. 2017). Vlessis et al. (2020) found that 55% of patients with solid tumors who had a somatic *BRCA1/2* PV also carried the same germline *BRCA1/2* PV. Hence, it is important to refer clinically relevant genes found on somatic testing to a cancer genetics service. The guidelines by ACMG serve as a useful guide for practicing clinicians. Diagnosis of a pathogenic germline variant has implications in further management not just for patients, but for predictive testing and management of their family members who may be at elevated risk for developing cancers.

Future clinical trials using *BRCA1/2* variant status or HRD status as an enrolment criterion may allow the indications of PARP inhibitors to be expanded beyond its current indications in *BRCA1/2*-associated cancer types. It is important for clinicians to ascertain somatic panel sequencing rather than relying solely on platinum sensitivity as a surrogate marker of HRD. The optimal choice and sequence of therapy in patients eligible for PARP inhibitor should also be studied. This will bring us closer to the goal of precision medicine with appropriate targeted therapies of low toxicity administered to genomically matched patients.

## METHODS

The specimen obtained from the epigastric lump was sent for *EGFR* mutation analysis by direct Sanger sequencing. Genomic DNA was extracted from the tissue sample. The extracted DNA then undergoes polymerase chain reaction (PCR) amplification for exons 18, 19, 20, and 21, and the following mutations screened for in the assay are shown in the Supplemental Materials. For *ALK* and *ROS1* mutation analysis, this was determined via interphase FISH. *ALK* break-apart, *ROS1* break-apart, *MET/CEP7* enumeration, and *RET* break-apart probes were performed on paraffin-embedded tissue. A total of 100 nonoverlapped nuclei were scored manually by two independent observers, and interpretation of the results was based on prevailing guidelines.

Peripheral blood was sent for *EGFR* T790M mutation. Isolation of cell-free DNA was performed using the Cobas cfDNA sample preparation kit. This was a real-time PCR assay for the semiquantification index (SQI) of mutations in exon 18 (G719X), deletion mutations in exon 19, T790M and S768I substitution mutations and insertion mutations in exon 20, and L858R and L861Q substitution mutations in exon 21 of the *EGFR* gene from serial collections of human plasma.

The patient's lung biopsy was sent for *EGFR* mutation analysis by Roche Cobas EGFR mutation test V2, a commercial real-time allele-specific PCR test. DNA is extracted from the tissue sample. The extracted DNA undergoes PCR amplification for exon 18, 19, 20, 21. The list of mutations that are targeted for by the assay is described in the Supplemental Materials.

The somatic tumor from the lung biopsy was sent for next generation sequencing via Oncomine Comprehensive Assay V3. Tumor tissue was obtained from archival formalin-fixed paraffin-embedded samples collected during the patient's lung biopsy. Genomic DNA and RNA from tumor were extracted from formalin-fixed paraffin-embedded or fresh-frozen tissue. A library was constructed using different panels, and the resulting amplicons were treated to partially digest, phosphorylate, and ligate to ion adapters with barcoding and purified. Quality and concentration of the libraries were determined using the Qubit 2.0 Fluorometer. Emulsion PCR and enrichment of template-positive Ion Sphere Particles, which contained clonally amplified DNA, were conducted using the Ion PGM OneTouch 2 system. Sequencing of this amplified DNA was subsequently performed on the Ion Torrent PGM sequencer. Data were analyzed primarily using Torrent Suite Variant Caller plugin, Ion Reporter software, and an in-house analysis pipeline v1.0.0 using Oncomine Reporter with reference genome hg19. The genomic analysis was developed to detect for somatic changes and was not designed or validated to interrogate for germline changes. Tumor mutation burden (TMB) was calculated based on the size of the panel and number of nonsynonymous mutations and mathematically approximated for 1 Mbp. The results of all molecular profiling were returned to the ordering clinician. The list of the genes analyzed from somatic tumor next-generation sequencing is found in the Supplemental Materials.

For the germline sequencing, the patient's DNA was extracted from peripheral blood and sent to Invitae. Genomic DNA was obtained from the blood and enriched for targeted regions using a hybridization-based protocol and sequenced using Illumina technology. Reads were aligned to a reference sequence (GRCh37), and sequence changes were identified and interpreted. Enrichment and analysis focus on the coding sequence of the indicated transcripts, 20 bp of flanking intronic sequence, and other specific genomic regions demonstrated to be causative of disease at the time of assay design. Promoters, untranslated regions, and other noncoding regions are not otherwise interrogated. All clinically significant observations are confirmed by orthogonal technologies, except individually validated variants and variants previously confirmed in a first-degree relative. Confirmation technologies include any of the following: Sanger sequencing, Pacific Biosciences SMRT sequencing, MLPA, MLPA-seq, Array CGH. The list of the genes analyzed from germline sequencing is found in Supplemental Materials. The germline testing panel had included evaluation of 59 genes for variants that are associated with genetic disorders, shown in Supplemental Materials.

## ADDITIONAL INFORMATION

### Data Deposition and Access

The variants and their interpretations have been submitted to ClinVar (https://www.ncbi.nlm.nih.gov/clinvar/) and can be found under accession number VCV000959846.3. Information can be made available upon reasonable request to the corresponding author.

### Ethics Statement

The study was approved by the Singhealth Centralized Review Board (CIRB 2021/2593). The patient provided his consent to publish his information and radiological images for this work. All procedures followed were in accordance with the Declaration of Helsinki.

### Acknowledgments

The authors thank our patient for sharing his presentation for this work.

### Author Contributions

All authors participated in the conception and design of the project, collection and assembly of data, data analysis and interpretation, and manuscript writing and final approval of manuscript. J.C. provided study materials or patients and administrative support. All authors are accountable for all aspects of the work.

### Competing Interest Statement

The authors have declared no competing interest.

### Referees

Benjamin H. Lok Anonymous

## Supplementary Material

Supplemental Material
