# Supplementary material for: Case report: olaparib use in metastatic lung adenocarcinoma with BRCA2 pathogenic variant
Source: Cold Spring Harb Mol Case Stud. 2022 Dec;8(7):a006223. doi: 10.1101/mcs.a006223 (PMC9808557; doi:10.1101/mcs.a006223)
Supplement: Supplemental Material [file supp_8_7_a006223__DC1.html]

Supplemental Material 

# Case report: olaparib use in metastatic lung adenocarcinoma with *BRCA2* pathogenic variant

## Supplemental Material

- Supplemental\_Material.pdf
