# Supplementary material for: Case report: olaparib use in metastatic lung adenocarcinoma with BRCA2 pathogenic variant
Source: Cold Spring Harb Mol Case Stud. 2022 Dec;8(7):a006223. doi: 10.1101/mcs.a006223 (PMC9808557; doi:10.1101/mcs.a006223)
Supplement: Supplemental Material [file supp_mcs.a006223_Supplemental_Material.pdf]

Supplementary Materials

List of EGFR mutations tested for on EGFR mutation analysis on the epigastric lump

Exon 18: E709K/Q/G/V/A/D
G719C / G719S / G719A
Exon 19: p.E746_A750del, p.E746_T751del, p.E746_T751delinsAla, p.E746_T751delinsIle, p.E746_T752delinsVal, p.E746_S752delinsAla, p.E746_S752delinsAsp, p.L747_E749del, p.L747_A750delinsPro, p.L747_T751delinsGln, p.L747_T751delinsPro, p.L747_T751delinsSer, p.L747_T751del, p.L747_S752del, p.L747_P753delinsGlu, p.L747_P753delinsSer, p.S752_I759del and other exon 19 deletions
Exon20: S768I (p.Ser768Ile)
T790M (p.Thr790Met)
Exon 20 insertions
Exon21: L858R (p.Leu858Arg)
L861Q (p.Leu861Gln)

List of EGFR mutations tested for on EGFR mutation analysis on primary lung tumor

Exon 18
c.2155G>A/T (p.G719X); c.2156G>C (p.G719X)
Exon 19 deletions
c.2235_2249del15 (p.E746_A750del); c.2236_2250del15 (p.E746_A750del);
c.2240_2257del18 (p.L747_P753>S); c.2240_2254del15 (p.L747_T751del);
c.2239_2256del18 (p.L747_S752del); c.2239_2251>C (p.L747_T751>P);
c.2237_2251del15 (p.E746_T751>A); c.2237_2255>T (p.E746_S752>V);
c.2239_2248TTAAGAGAAG>C (p.L747_A750>P); c.2239_2253del15 (p.L747_T751del);
c.2239_2247del9 (p.L747_E749del); c.2235_2252>AAT (p.E746_T751>I);
c.2236_2253del18 (p.E746_T751del); c.2237_2254del18 (p.E746_S752>A);
c.2238_2255del18 (p.E746_S752>D); c.2238_2248>GC (p.L747_A750>P);
c.2238_2252>GCA (p.L747_T751>Q); c.2239_2258>CA (p.L747_P753>Q);
c.2240_2251del12 (p.L747_T751>S); c.2233_2247del15 (p.K745_E749del);
c.2253_2276del24 (p.S752_I759del); c.2235_2248>AATTC (p.E746_A750>IP);
c.2237_2252>T (p.E746_T751>V); c.2235_2251>AATTC (p.E746_T751>IP);
c.2235_2255>AAT (p.E746_S752>I); c.2237_2253>TTGCT (p.E746_T751>VA);
c.2237_2257>TCT (p.E746_P753>VS); c.2238_2252del15 (p.L747_T751del);
c.2239_2256>CAA (p.L747_S752>Q);

Exon 20
c.2303G>T (p.S768I); c.2369C>T (p.T790M);
c.2307_2308ins9 (p.A767_V769dup); c.2309_2310AC>CCAGCGTGGAT (p.V769_D770insASV); c.2310_2311insGGT (p.D770_N771insG);
c.2311_2312ins9 (p.D770_N771insSVD);
c.2319_2320insCAC (p.H773_V774insH)

Exon 21
c.2573T>G (p.L858R); c.2573_2574TG>GT (p.L858R)
c.2582T>A (p.L861Q)

List of Genes analyzed from next generation sequencing of primary lung tumor with Oncomine Comprehensive Assay V3

Hotspot:
AKT1, AKT2, AKT3, ALK, AR, ARAF, AXL, BRAF, BTK, CBL, CCND1, CDK4, CDK6, CHEK2, CSF1R, CTNNB1, DDR2, EGFR, ERBB2, ERBB3, ERBB4, ERCC2, ESR1, EZH2, FGFR1, FGFR2, FGFR3, FGFR3, FLT3, FOXL2, GATA2, GNA11, GNAQ, GNAS, H3F3A, HIST1H3B, HNF1A, HRAS, IDH1, IDH2, JAK1, JAK2, JAK3, KDR, KIT, KNSTRN, KRAS, MAGOH, MAP2K1, MAP2K2, MAP2K4, MAPK1, MAX, MDM4, MED12, MET, MTOR, MYC, MYCN, MYD88, NFE2L2, NRAS, NTRK1, NTRK2, NTRK3, PDGFRA, PDGFRB, PIK3CA, PIK3CB, PPP2R1A, PTPN11, RAC1, RAF1, RET, RHEB, RHOA, ROS1, SF3B1, SMAD4, SMO, SPOP, SRC, STAT3, TERT, TOP1, U2AF1 and XPO1

Full length:
ARID1A, ATM, ATR, ATRX, BAP1, BRCA1, BRCA2, CDK12, CDKN1B, CDKN2A, CDKN2B, CHEK1, CREBBP, FANCA, FANCD2, FANCI, FBXW7, MLH1, MRE11A, MSH2, MSH6, NBN, NF1, NF2, NOTCH1, NOTCH2, NOTCH3, PALB2, PIK3R1, PMS2, POLE, PTCH1, PTEN, RAD50, RAD51, RAD51C, RAD51D, RAD51B, RB1, RNF43, SETD2, SLX4, SMARCA4, SMARCB1, STK11, TP53, TSC1 and TSC2

Copy Number:
AKT1, AKT2, AKT3, ALK, AR, AXL, BRAF, CCND1, CCND2, CCND3, CCNE1, CDK2, CDK4, CDK6, EGFR, ERBB2, ESR1, FGF19, FGF3, FGFR1, FGFR2, FGFR3, FGFR4, FLT3, IGF1R, KIT, KRAS, MDM2, MDM4, MET, MYC, MYCL, MYCN, NTRK1, NTRK2, NTRK3, PDGFRA, PDGFRB, PIK3CA, PIK3CB, PPARG, RICTOR, TERT

Fusion Drivers:
AKT2, ALK, AR, AXL, BRAF, BRCA1, BRCA2, CDKN2A, EGFR, ERBB2, ERBB4, ERG, ESR1, ETV1, ETV4, ETV5, FGFR1, FGFR2, FGFR3, FGR, FLT3, JAK2, KRAS, MDM4, MET, MYB, MYB1, NF, NOTCH1, NRG1, NTRK1, NTRK2, NTRK3, NUTM1, PDGFRA, PDGFRB, PIK3CA, PPARG, PRKACA, PRKACB, PTEN, RAD51B, RAF1, RB1, RELA, RET, ROS1, RSPO2, RSPO3, TERT

List of Genes analyzed from germline sequencing of peripheral blood by Invitae
The tests performed included the Invitae Common Hereditary Cancers Panel, Invitae Breast and Gyn Cancers panel, Add-on preliminary-evidence genes for Breast and Gyn Cancer .

The list of the genes include:
ABRAXAS1, AKT1, APC, ATM*, AXIN2, BARD1, BMPR1A, BRCA1, BRCA2, BRIP1, CDC73, CDH1, CDK4, CDKN2A (p14ARF), CDKN2A (p16INK4a), CHEK2, CTNNA1, DICER1*, EGFR, EPCAM*, FANCC, FANCM, GREM1*, HOXB13, KIT, MEN1*, MET*, MLH1*, MRE11, MSH2*, MSH3*, MSH6*, MUTYH, NBN, NF1*, NTHL1, PALB2, PDGFRA, PIK3CA, PMS2*, POLD1*, POLE, PTEN*, RAD50, RAD51C, RAD51D, RECQL*, RINT1, SDHA*, SDHB, SDHC*, SDHD, SMAD4, SMARCA4, STK11, TP53, TSC1*, TSC2, VHL, XRCC2*

An asterik indicates that this gene has a limitation.

Based on validation study results, this assay achieves >99% analytical sensitivity and specificity for single nucleotide variants, insertions and deletions <15bp in length, and exon-level deletions and duplications. Invitae's methods also detect insertions and deletions larger than 15bp but smaller than a full exon but sensitivity for these may be marginally reduced. Invitae’s deletion/duplication analysis determines copy number at a single exon resolution at virtually all targeted exons. However, in rare situations, single-exon copy number events may not be analyzed due to inherent sequence properties or isolated reduction in data quality. Certain types of variants, such as structural rearrangements (e.g. inversions, gene conversion events, translocations, etc.) or variants embedded in sequence with

complex architecture (e.g. short tandem repeats or segmental duplications), may not be detected. Additionally, it may not be possible to fully resolve certain details about variants, such as mosaicism, phasing, or mapping ambiguity. Unless explicitly guaranteed, sequence changes in the promoter, non-coding exons, and other non-coding regions are not covered by this assay. Please consult the test definition on our website for details regarding regions or types of variants that are covered or excluded for this test. This report reflects the analysis of an extracted genomic DNA sample. In very rare cases (such as circulating hematolymphoid neoplasm, bone marrow transplant, recent blood transfusion, or maternal cell contamination), the analyzed DNA may not represent the patient's constitutional genome.
MSH3: Sequencing analysis of the repeat region of exon 1 (5:79950697-79950765) is not offered RECQL: Sequencing analysis for exons 3, 5, 15 includes only cds +/- 10 bp. MET: Sequencing analysis for exons 12 includes only cds +/- 10 bp. MSH6: Sequencing analysis for exons 7, 10 is limited to cds +/-10 bp. NF1: sequencing analysis for exons 2, 7, 25, 41, 48 includes only cds +/- 10 bp. GREM1: Promoter region duplication testing only. SDHA: Deletion/ duplication analysis is not offered for this gene and sequencing analysis is not offered for exon 14. Sequencing analysis for exons 6-8 includes only cds +/- 10 bp. MSH2: Analysis includes the exon 1-7 inversion (Boland mutation). Sequencing analysis for exons 2, 5 includes only cds +/- 10 bp. TSC1: Sequencing analysis for exons 21 includes only cds +/- 10 bp. ATM: Sequencing analysis for exons 6, 24, 43 includes only cds +/- 10 bp. PMS2: Sequencing analysis for exons 7 includes only cds +/- 10 bp. POLD1: Sequencing analysis for exon 22 is limited to cds +/-10 bp. MEN1: Sequencing analysis for exon 2 is limited to cds +/-10 bp. MLH1: Sequencing analysis for exon 12 is limited to cds +/-10 bp. DICER1: Sequencing analysis for exons 22 includes only cds +/- 10 bp. PTEN: Sequencing analysis for exons 8 includes only cds +/- 10 bp. SDHC: Sequencing analysis for exons 2, 6 includes only cds +/- 10 bp. EPCAM: Sequencing analysis is not offered for this gene. APC: Sequencing analysis for exon 5 is limited to cds +/-10 bp.
